# Osteoarthritis of the sternoclavicular joint: is clavicular length a risk factor?

**DOI:** 10.1007/s00402-025-05967-w

**Published:** 2025-06-30

**Authors:** Aditya Vadgaonkar, Ali Darwich, Sascha Gravius, Michael Hackl, Johann Rink, Sonja Janssen, Tobias Baumgärtner, Udo Obertacke

**Affiliations:** 1 Department of Orthopaedic and Trauma Surgery, University Medical Centre Mannheim, Medical Faculty Mannheim, University of Heidelberg, Theodor-Kutzer-Ufer 1-3, 68167 Mannheim, Germany; 2 Department of Radiology and Nuclear Medicine, University Medical Centre Mannheim, Medical Faculty Mannheim, Heidelberg University, Theodor-Kutzer-Ufer 1-3, 68167 Mannheim, Germany; 3 Meliva MVZ Westpfalz Radiologie Kaiserslautern Lutrinastr, 27 67655 Kaiserslautern, Germany

**Keywords:** Sternoclavicular joint, Osteoarthritis, Risk factors, Biomechanics, Shoulder joint

## Abstract

**Introduction:**

Osteoarthritis (OA) is the most prevalent disorder of the Sternoclavicular Joint (SCJ), with a reported lifetime prevalence of approximately 50%, yet it is rarely observed in individuals under the age of 35. When symptomatic, it manifests with pain and swelling. Although OA is strongly age-related, the influence of biomechanical factors - such as clavicular length - remains unknown.

**Materials and methods:**

In this retrospective case-control study, computed tomography (CT) scans of adult polytrauma patients admitted to our hospital between 2012 and 2014 were evaluated. OA was defined radiologically by the presence of osteophytes, subchondral cysts, or cortical sclerosis, and a score from 0 to 6 was assigned according to the severity of these changes. Medial-most and lateral-most points of the clavicle were used to determine clavicular length. The x-, y-, and z-coordinates of these two points were extracted from the DICOM metadata and clavicular length was calculated as the distance between these two points using 3D geometry.

**Results:**

A total of 334 SCJs from 167 patients (36% female, mean age 48.5 ± 20.5 years) were analyzed. Mean clavicular length was shorter in the group with OA (144 ± 11 mm vs. 150 ± 12 mm, *p* < 0.001, right; 146 ± 11 mm vs. 153 ± 11 mm, *p* < 0.001, left). Age and clavicular length were independent risk factors on multivariate regression model. The logistic model showed a robust discriminative ability with the area under the curve being 90% (right) and 91% (left). Adjusted odds ratios were 0.93 (right) and 0.92 (left). Mean clavicular length showed a decreasing trend with increasing OA scores (*p* = 0.01).

**Conclusions:**

Our findings suggest that shorter clavicles are associated with a higher prevalence of sternoclavicular joint osteoarthritis, demonstrating an inverse correlation between clavicular length and the severity of the radiological signs of degeneration. Prospective studies are warranted to further investigate the clinical implications of clavicular shortening and its potential role in the development of SCJ osteoarthritis.

## Introduction

As the only bony connection tethering the axial skeleton to the upper appendicular skeleton, the clavicle functions as a dynamic strut transmitting forces from the centre to the periphery [1]. Medially the clavicle articulates with the superolateral portion of the manubrium forming the sternoclavicular joint (SCJ). Anatomically, the SCJ is a diarthrodial joint and mechanically it is analogue to a spherical bearing allowing for three degrees of freedom with restricted translational movements but preserved rotational capacity (elevation-depression, protraction-retraction, and axial rotation) [2]. The articulating surfaces between the sternum and the clavicle are relatively asymmetrical resulting in high pressure in a small contact area [3, 4]. This mechanical situation, while permitting for a high degree of mobility, predisposes the SCJ to more wear and tear, possibly resulting in a higher prevalence of osteoarthritis (OA). It is therefore unsurprising that OA is the most common disorder affecting this joint [5]. Although not frequently symptomatic, it can present with chest pain or swelling [4]. 

Diagnosis of OA usually relies on radiographic imaging with computed tomography (CT) demonstrating superior capability in detecting and characterizing OA-related changes as compared to conventional radiographs [6, 7]. Epidemiological data indicate that the prevalence of OA at the SCJ increases significantly with age, affecting over 90% of individuals over 70 years [7–11]. While advancing age consistently correlates positively with the prevalence of OA, such a consensus regarding the role of other factors such as gender and side affected does not exist [6, 10, 12, 13]. Notably, the influence of biomechanical factors like clavicular length on OA prevalence has not been thoroughly investigated.

The Delft Shoulder and Elbow Model (DSEM) represents a three-dimensional biomechanical model of the upper extremity that treats the shoulder girdle as a closed kinematic chain, consisting of clavicle, thorax, scapula, and humerus [14]. When employing this model to calculate the torques exerted on the joints during shoulder movements, it becomes apparent that the length of the clavicle plays a pivotal role in determining the distribution of torque across the joints. Reduction in the length of the clavicle after a fracture is associated with functional impairments in shoulder activity, manifesting as diminished muscular strength, muscle atrophy, decreased range of motion and discomfort with movement [15]. Additionally, Teubner et al. suggested that decreased clavicular length could intensify forces acting on the SCJ [2]. It is therefore undisputed that length of the clavicle is an important biomechanical feature of the shoulder girdle system. Despite this recognised importance of clavicular length in shoulder mechanics, its influence on the aetiology of OA at the SCJ remains unclear. This motivated our retrospective case-control study, which aimed to investigate patient-specific and biomechanical factors that might predispose individuals to SCJ OA. We hypothesized that clavicular length significantly influences the presence of SCJ OA by altering force transmission dynamics at the joint.

## Materials and methods

### Study design and setting

This retrospective case-control study was carried out on individuals brought to the emergency department of a tertiary-level trauma centre between 2012 and 2014 with severe or multiple injuries. CT imaging was performed using Siemens CT scanners: Somatom Force (2 × 192 slice dual-source CT), Somatom Definition Flash (2 × 128 slice dual-source CT) and Emotion 16 (16-slice single-source CT) (Siemens Healthcare GmbH, Erlangen, Germany). Imaging was conducted using a helical technique with collimations of 0.6–1.2 mm, pitch factors ranging from 0.55 to 3. Reconstructions were performed with slice thickness ≤ 1.5 mm. Due to the diverse indications for the included CT scans, the scan range along the z-axis varied significantly, resulting in a Volume CT Dose Index (CTDIvol) range of 0.18 to 74.89 mGy. Reconstructed image data were stored in Digital Imaging and Communications in Medicine (DICOM) format within the picture archiving and communication system (PACS). All measurements were performed on the Aycan Workstation OsiriX (Version 4.0, Aycan Digitalsysteme, Würzburg, Germany).

### Inclusion and exclusion criteria

All adult patients fulfilling the Grade A or B recommendation criteria laid out as per the *Level 3 guideline on the treatment of patients with severe/multiple injuries* published by the national trauma society [16] were included in the study. This includes patients with suspected severe injury, based on prehospital triage criteria such as reduced consciousness, hemodynamic instability, or high-risk mechanisms of injury. Patients were excluded when they had any radiographic signs of acute or previous clavicular trauma or pathology including neoplasia, infection, deformity or were under the age of 18. None of the included CT scans was done for the sole purpose of the study.

### Procedure

Measurements were performed with a pseudonymized CT scan database kept in Microsoft Excel (Microsoft Corporation, Redmond, WA, US) using Aycan DICOM-Viewer on a monitor approved for radiological reporting. The CT- images were set to a bone window, and a board-certified orthopaedic surgeon (UO) performed the initial review to assess study eligibility based on inclusion and exclusion criteria. Each case was then independently analysed by the first author (AV) on two separate occasions, with a minimum interval of two months between readings. For each session, the axial, coronal, and sagittal reconstructions were evaluated to assign a score of 0–2 based on the severity of radiological changes in the SCJ (as detailed in Table 1) and the final OA score was the sum of these values. The length was calculated on two separate occasions and the final values used for subsequent analyses were the mean of the two measurements. To assess interrater reliability, 20 randomly selected cases were independently evaluated by a second orthopaedic surgeon (UO), blinded to the original readings. To minimise interobserver variability, viewing of the scans was standardised by using the same computer console under similar settings. The age and gender of the patient was determined from the patient data.

### Definitions

**Fig. 1:**
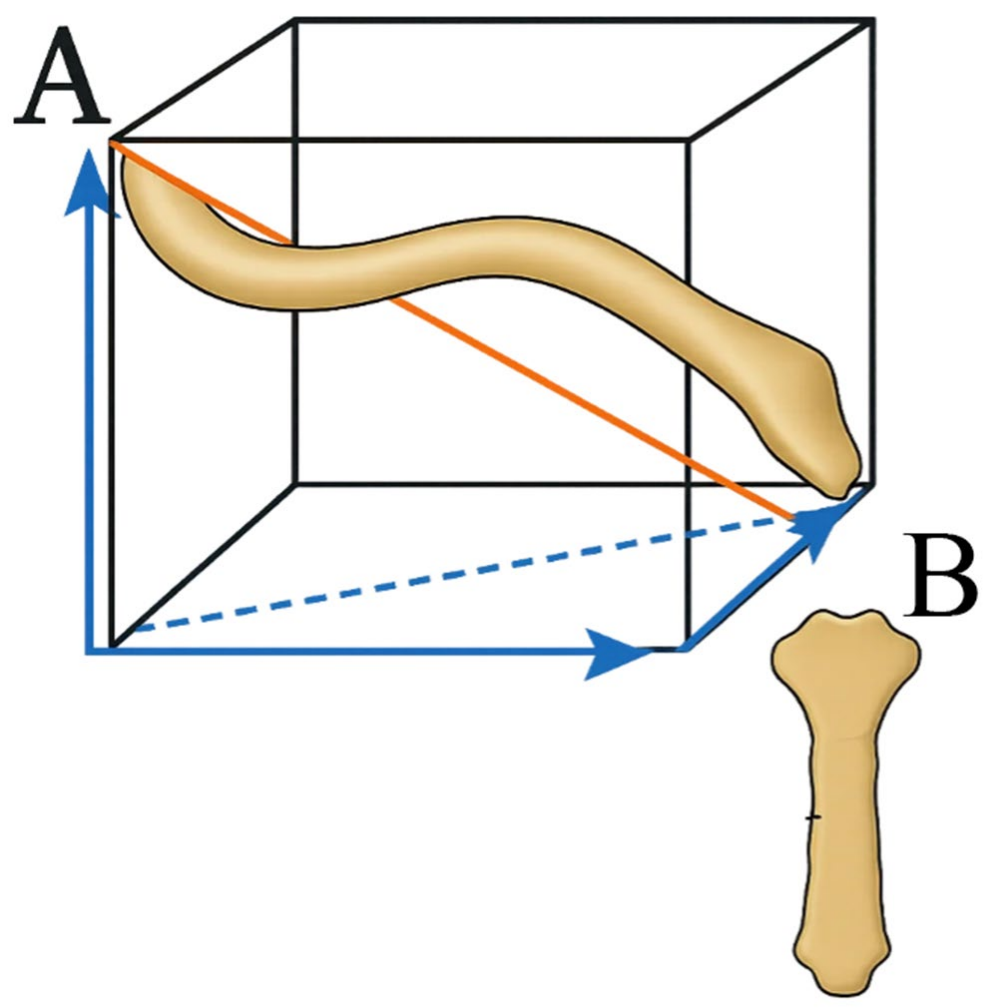
3D measurement of the length of the clavicle using Euclidean Formula. Point A = Acromioclavicular Joint, Point B = Sternoclavicular Joint. Distance AB is the length of the Clavicle

Clavicular length was defined as the straight-line distance between the medial-most point of the clavicle in the SCJ and the lateral-most point of the clavicle on the acromioclavicular joint (ACJ) as seen on axial view in the CT scan. The medial-most and lateral-most points of the clavicle were marked on the slices passing through the SCJ and ACJ respectively. The x-, y-, and z-axis coordinates of these two points were determined from the DICOM metadata and subsequently used to calculate clavicular length as the distance between these two points using the Euclidean formula [17] (Fig. 1):

$${\rm{d}} = \sqrt {{{\left( {{x_a} - {x_b}} \right)}^2} + {{\left( {{y_a} - {y_b}} \right)}^2} + {{\left( {{z_a} - {z_b}} \right)}^2}} $$

where x_a_ is the x coordinate of Point A, y_a_ is the y coordinate of Point A and z_a_ is the z coordinate of Point A. Similarly for Point B.

**Fig. 2:**
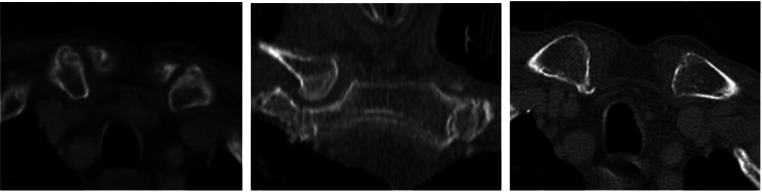
Subchondral cyst, subchondral sclerosis, osteophyte formation (left to right)

The sternoclavicular joints were examined for osteoarthritic changes following the Kellgren and Lawrence [18] criteria, at both the sternal and clavicular terminations of the joint. The presence of cortical sclerosis, subchondral cyst or osteophyte formation was considered indicative for osteoarthritis (Fig. 2). For each of these parameters, a score 0, 1 or 2 was assigned depending on the severity of the changes (as mentioned in Table 1). The score of each of the parameter was then added to get a final OA score. A score of 1 or above was considered to be indicative of OA.

**Table 1:** OA score based on the radiological severity of the changes

Score	Cortical Sclerosis	Subchondral cyst	Osteophyte formation
0	Absent	Absent	Absent
1	Involvement of < 50% of the joint surface	2 or fewer cysts	Size < 0.5 mm
2	Involvement of > 50% of the joint surface	More than 2 cysts	Size > 0.5 mm

### Data analysis

This study has been reported in line with the STROBE Statement [19]. Mean values and standard deviations (SD) were calculated to describe quantitative morphometric parameters. Intrarater and interrater reliability for clavicular length measurements were assessed using the intraclass correlation coefficient (ICC). A two-way mixed effects model was used to determine interrater reliability, while intrarater reliability was evaluated using repeated measurements by a single rater over time. Normal distribution of the continuous variables such as clavicular length and age was assessed using he Shapiro-Wilk test and visual inspection of histograms.The relationship between patient characteristics (age, gender, clavicular length) and the presence of OA at the SCJ as the outcome variable was analysed using t-tests, with the threshold for type I error set at 0.05. A multivariate logistic regression analysis was performed to evaluate the combined influence of these factors. Differences in mean clavicular lengths across groups with varying OA scores were assessed using multivariate analysis of variance (ANOVA). Subgroup analysis for unilateral SCJ OA was conducted using the binomial test to evaluate side-specific prevalence. All statistical analyses were carried out using GraphPad Prism (version 9.3.1 for MacOS, GraphPad Software LLC).

### Ethics approval

This study was approved by the Ethics Committee of Clinical Research at our institution (Ethics Committee II, University Medical Centre Mannheim, Medical Faculty Mannheim, Heidelberg University, Theodor-Kutzer-Ufer 1–3, 68167, Mannheim, Approval 2016-870R-MA) and conducted in accordance with local ethical standards and the principles outlined in the 1964 Helsinki Declaration and its subsequent amendments. Individual consent was not required, as all patient data were collected retrospectively and anonymized prior to analysis.

## Results

A total of 334 SCJs from 167 patients (64% males, 36% females) with a mean age of 48.5 ± 20.5 years were analysed on axial, coronal and sagittal CT scans to look for presence of osteoarthritis using the criteria as mentioned earlier. Intrarater reliability demonstrated excellent agreement (interclass coefficient of correlation, ICC right 0.90; left 0.92) whereas interrater reliability, determined using a two-way mixed effects model across different raters, also showed good agreement (ICC of 0.84 right; 0.86 left).

### Length and asymmetry of the clavicle

The length of the clavicle ranged between 119 mm and 179 mm with the mean length of the right side significantly shorter than that of the left (147 ± 12 mm vs. 150 ± 12 mm, *p* = 0.02). The mean difference in the length of left and the right clavicle was 2.9 ± 1.3 mm. 17 subjects (10.2%) had a difference of more than 10 mm between the two sides.

### Length of clavicle and gender

The length of the clavicle was significantly shorter in females as in males in both, the right (138 ± 9 mm vs. 152 ± 10 mm, *p* < 0.001) and the left side (141 ± 9 mm vs. 155 ± 10 mm, *p* < 0.001).

### Age and prevalence of OA

**Fig. 3:**
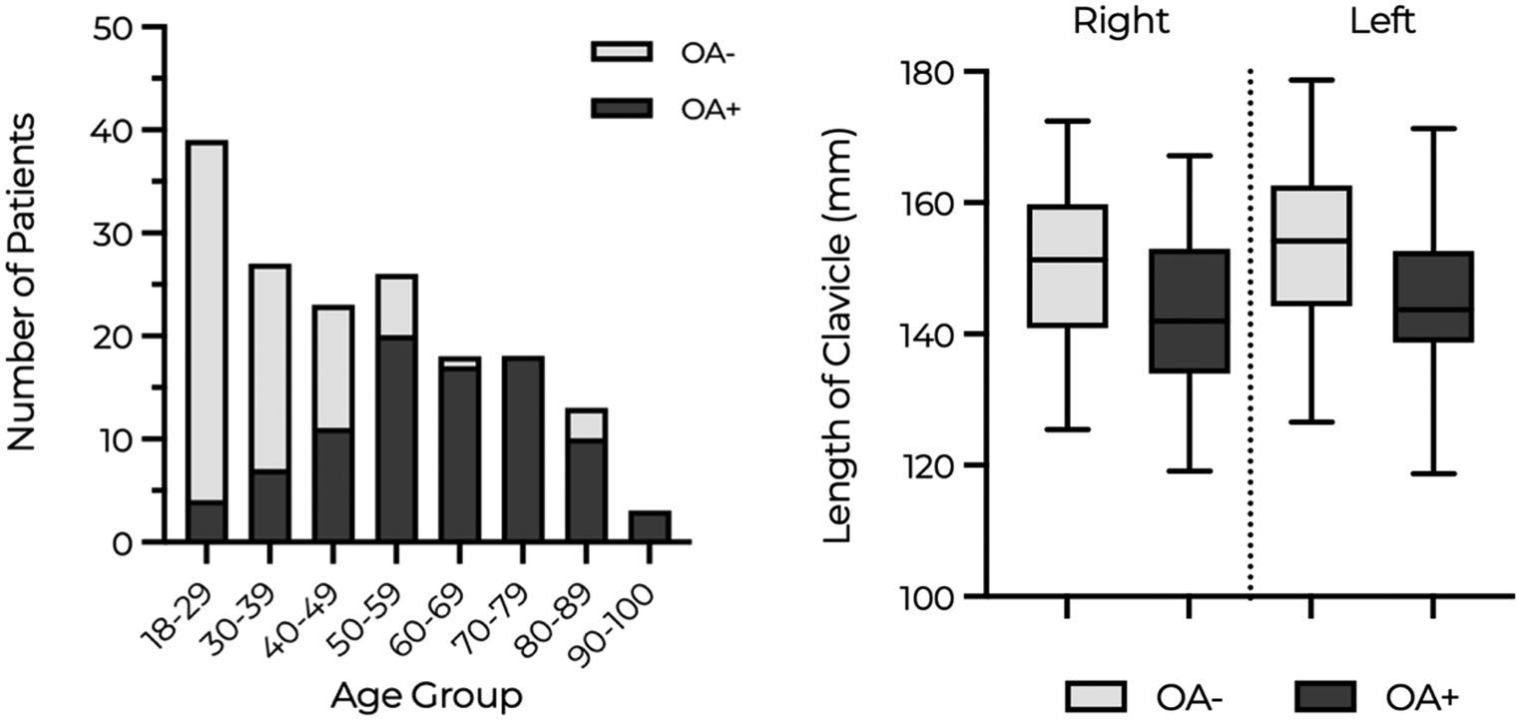
**A**: Bar chart showing the distribution of patients (OA + and OA-) across age groups (X-axis) with the number of patients (Y-axis). **B**: Box-and-whisker plot comparing the lengths of the clavicle on two sides. The thick horizontal line in the middle of each box represents the mean clavicle length. The whiskers indicate the maximum and minimum values. The box represents the interquartile range (*p* < 0.001 right; < 0.001 left)

A total of 167 patients with 334 SCJs were evaluated, among which 90 patients (53.9%) and 155 joints (46.40%) exhibited radiological evidence of OA. The prevalence of OA demonstrated a direct correlation with age, with nearly 100% of the patients aged 60 or older displaying at least one radiological sign for OA. The distribution of OA with age is graphically presented below (Fig. 3a). Furthermore, a comparative analysis of the mean age between the group with and without OA on at least one side using t-test revealed a highly significant statistical difference (61 years for OA + and 34 years for OA-, *p* < 0.001).

### Length of clavicle and prevalence of OA

Comparing the mean clavicular length using t-test showed a significantly shorter clavicle in the group with OA as compared to the group without OA (146 ± 11 mm vs. 153 ± 11 mm, *p* < 0.001, left and 144 ± 11 mm vs. 150 ± 12 mm, p = < 0.001, right). (Fig. 3b)

### Length of clavicle and OA score

**Fig. 4:**
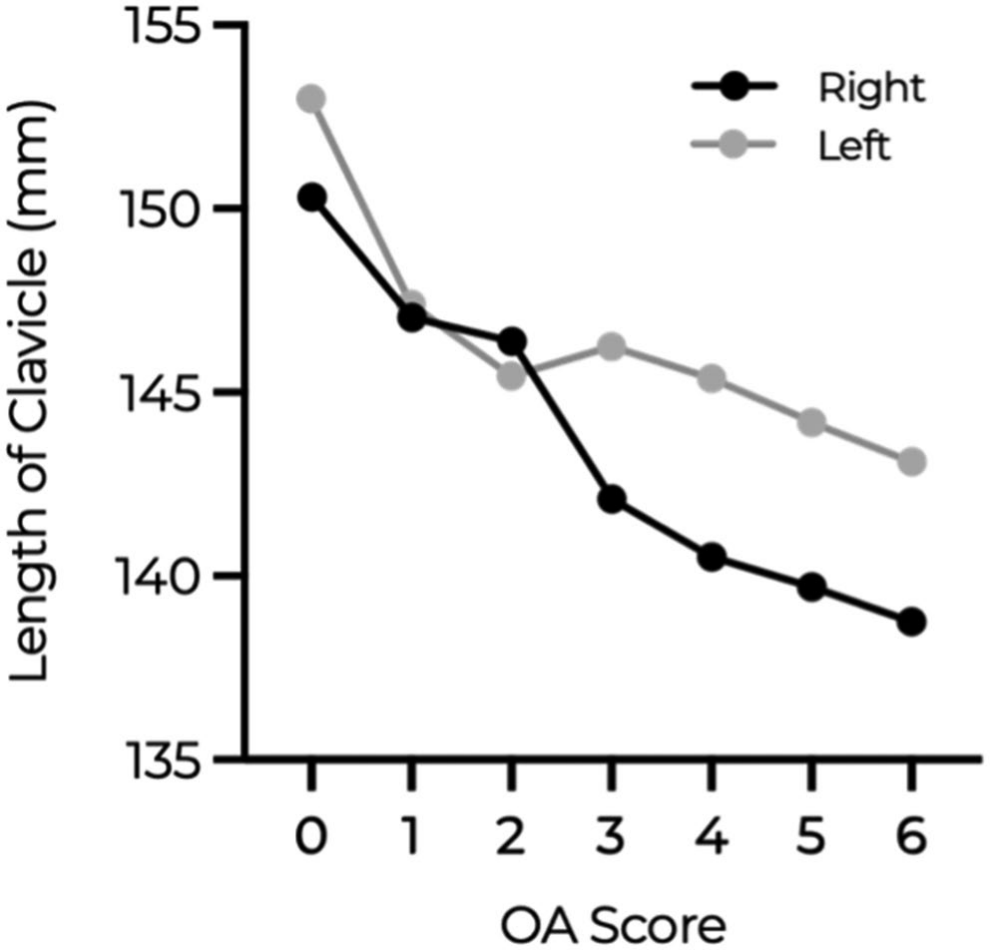
Graph showing the relationship between clavicle length (Y-axis) and OA score (X-axis)

The results of the one-way ANOVA comparing the mean clavicular lengths across different OA score groups are depicted in Fig. 4. The analysis revealed a statistically significant difference in mean clavicular length among the various OA score categories (*p* = 0.01), showing a clear trend of decreasing mean clavicular length with increasing OA scores.

### Prevalence of OA with side and gender

OA tended to be present more often on the right side than the left side, however, without reaching statistical significance (50.9% right vs. 41.9% left, *p* = 0.12). In the study population, 56 males (52%) while 34 females (56%) had radiologically detectable OA. This, however, also did not reach statistical significance (*p* = 0.63).

### Presence of unilateral osteoarthritis

Out of the 90 patients with OA of the SCJ, 65 presented with bilateral OA, while 25 had unilateral involvement. Among those with unilateral OA, 18 cases (72%) occurred on the shorter clavicle, and 7 (28%) occurred on the longer clavicle. A binomial test comparing this distribution showed that the difference was statistically significant (*p* = 0.02), indicating that OA is more likely to develop on the shorter clavicle of the same subject in unilateral cases, rather than being equally distributed between the shorter and longer sides.

### Multivariate logistic regression model

Using age, clavicular length and gender as independent variables and presence of osteoarthritis as the outcome variable, we created a multivariate regression model. This revealed that age and clavicular length were independent risk factors for OA while gender did not reach statistical significance. The results of the model are summarised in the table below (Table 2) The final logistic model illustrated robust discriminative ability with area under the curve being 90% on the right and 91% on the left.

**Table 2:** Multivariate logistic regression. aOR: adjusted odds ratio, CI: confidence interval, ROC: Receiver-operating characteristic

	Right	Left
**Age** ^****^		
aOR	1.11	1.11
95% CI	1.07–1.14	1.08–1.15
p	< 0.0001	< 0.0001
**Clavicular Length** ^***^		
aOR	0.93	0.92
95% CI	0.88–0.97	0.87–0.96
p	0.003	0.0008
**Gender**		
aOR	2.26	1.71
95% CI	0.71–7.50	0.54–5.55
p	0.16	0.36
**ROC Curve**		
Area	0.90	0.91
95% CI	0.85–0.94	0.86–0.96
p	< 0.0001	< 0.0001
R^2^	0.50	0.52

## Discussion

Osteoarthritis is by far the most common disorder affecting the sternoclavicular joint with epidemiological data suggesting that its lifetime prevalence is well over 50% [10]. Despite its high prevalence, factors influencing the development of OA beyond increasing age remain poorly understood. Biomechanical studies have highlighted the role of clavicular length in determining the torque exerted at the sternoclavicular joint, suggesting a potential link between clavicle length and joint stress [2, 15]. Building on this, we hypothesized that clavicular length may also play a significant role in the risk of developing OA at the SCJ and through our study, we were able to show a negative association between the length of the clavicle and the severity of OA.

For the radiological diagnosis of OA, we looked for the presence of cortical sclerosis, subchondral cyst and osteophyte formation [20]. Given that the reliability of joint space reduction as a marker of OA at the SCJ is debated because of its non-weight-bearing nature, it was excluded from our diagnostic criteria [9]. Previous studies on the knee and hip joints have established a positive association between the number of cysts, size of the osteophytes, the degree of sclerosis and the severity of osteoarthritis [21, 22]. We adapted this to the SCJ to rate the severity of osteoarthritic changes with a maximum score of 6. Using this new OA score for the diagnosis, we were able to confirm a clear positive association between age and the prevalence of OA, consistent with previous studies [7–9, 11]. Additionally, we also observed a progressive increase in OA score with advancing age. This positive association between age and OA is however unsurprising. While mechanically this can be explained by increased wear and tear, recent biochemical studies have showed that degradation of cartilage with age due to increased activity of matrix metalloproteinases (MMPs) as well as release of pro-inflammatory cytokines such as interleukin 1 (IL-1) and tumour necrosis factor alpha (TNF α) causes cartilage degradation and contributes to the progression of OA [23–25]. 

In previous studies, gender as a risk factor for the development of OA has been extensively studied. According to a systematic review by Tschon et al., females have a higher risk, attributable to a smaller joint surface area, lower muscle mass, and reduced cartilage volume, all of which increase the mechanical stress on the joints leading to OA [26]. This has been shown to be especially true in weight bearing joints such as the hip and the knee joint [27]. Some studies have established that these gender-based differences also extend to the SCJ [6, 13]. However, our research along with others found no statistically significant difference in the prevalence of OA at the SCJ between the two genders [10, 12]. This can however be well explained by the anatomy of the SCJ. The SCJ has been shown to have a relatively uniform surface across genders [28]. Consequently, the force transmission and load distribution on the joint are similar for both genders, resulting in no significant differences in mechanical stress and hence no difference in OA prevalence.

We speculated that length is an inherent characteristic of the clavicle that influenced OA at the SCJ. We employed coordinate geometry to calculate the clavicular length, enabling a 3D assessment that considered both the clavicle’s depth in the axial plane as well as its elevation in the coronal plane. This approach contrasts with other studies that measured clavicular length as a linear distance between SCJ and ACJ [29]. Our multivariate regression model using age, clavicular length and gender as independent variables as presence of OA as the outcome variable had a robust discriminative ability and returned age and clavicular length as statistically significant variables.

Interestingly, our findings diverge from patterns observed in other joints such as the knee, where longer bones have an elevated risk of developing OA, presumable because of increased torque acting on the joint [30, 31]. Experimental cadaveric study by Teubner et al. can be used to explain this apparent discrepancy, which demonstrated that a 1 cm shortening of the clavicle leads to a 40% increase in force transmission through the SCJ [2]. Hillen et al. further demonstrated that a shorter clavicle alters resting position and movement patterns through increased protraction and anterior rotation, causing scapular position-offset and changing the orientation of both the ACJ and SCJ. These kinematic changes likely contribute to increased torque and biomechanical stress at the SCJ, promoting osteoarthritis development [32]. 

Our findings indicate that clavicular length plays a contributory role in the development of OA at the SCJ, likely due to its biomechanical influence on force transmission within the shoulder girdle. This observed association prompts further consideration in clinical scenarios where clavicular shortening occurs, such as after fractures. Although causality cannot be inferred from our data, it remains plausible that post-traumatic shortening could similarly alter joint mechanics and potentially increase the risk of SCJ osteoarthritis over time. It remains uncertain whether shorter clavicles predispose to OA or if degenerative changes contribute to clavicular shortening over time. However, the absence of a correlation between clavicle length and age in our data argues against the latter. Further prospective studies are needed to clarify the temporal and causal nature of this relationship.

### Limitations

Our study has certain limitations that warrant consideration. First, as it was conducted using CT scans from patients with severe or multiple injuries, the sample population may not be representative of the general population, potentially introducing selection bias. Second, we lacked data on the presence of osteoarthritis symptoms. Since not all individuals with radiological signs of OA experience symptoms, the proportion of our study population with SCJ-related complaints remains unclear. Third, we did not have information on patient handedness. Additionally, patient height was not recorded in our dataset. Taller individuals may tend to have longer clavicles, introducing a potential confounding factor in our analysis of clavicle length as a risk factor for OA. Also, the retrospective nature of our study limits our ability to establish causative relationships. Finally, our analysis did not include patients with post-traumatic clavicular shortening, and thus our findings are limited to naturally occurring variations in clavicle length.

## Conclusion

In conclusion, our study demonstrates a strong relationship between clavicular length and the presence of OA at the SCJ, with shorter clavicles being associated with a higher prevalence of radiographic OA. We speculate that this association may be explained by altered biomechanics and increased joint loading in shorter clavicles. While our findings are based on naturally occurring anatomical variation, they raise the possibility that post-traumatic clavicular shortening—such as after fracture—could similarly impact SCJ mechanics and contribute to joint degeneration. Further biomechanical and prospective clinical studies are needed to explore this relationship and to assess its potential relevance in the context of fracture management.

## Data Availability

Data is provided within the manuscript.
